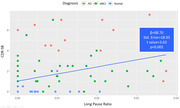# Long Pause Ratio in Speech Reflecting Dementia Severity and Hippocampal Atrophy in Early Alzheimer's Disease

**DOI:** 10.1002/alz.086309

**Published:** 2025-01-03

**Authors:** Bo‐Xiang Lin, Chia‐Ju Chou, Chih‐Ting Chang, Chia‐Ying Lee, Yi‐ Fang Chuang, Yen‐Ling Chiu, Yi‐Chien Liu

**Affiliations:** ^1^ Fu Jen Catholic University, New Taipei City, Taiwan Taiwan; ^2^ Cardinal Tien Hospital, New Taipei City Taiwan; ^3^ National Taipei University of Nursing and Health Sciences, Taipei Taiwan; ^4^ Institute of Linguistics, Academia Sinica, Taipei Taiwan; ^5^ National Yang Ming Chiao Tung University, Taipei, NA Taiwan; ^6^ Far‐Eastern Memorial Hospital, New Taipei Taiwan

## Abstract

**Background:**

It has been found that speech analysis is a sensitive method for early Alzheimer’s disease (AD) detection. Among the various linguistic features, silent pause has emerged as a fair indicator, exhibiting higher prevalence in individuals with AD compared to general population. This study aimed to further examine the relationship between long pause ratio (LPR) in speech and traditional markers of disease progression, like biomarkers, neuroimaging features and neuropsychological tests.

**Method:**

Since 2021 to 2023, we recruited 104 participants from memory clinic of Cardinal Tien hospital, Taipei, Taiwan. All participants received amyloid PET, 3.0 T brain MRI, and comprehensive neuropsychological tests. The determination of amyloid positivity came from consensus visual rating and quantitative methods utilizing SUVR (standardized uptake value ratio). Speech samples were collected using the picture description test, in which the participants describe three Taiwanese‐culture‐based pictures in one minute. LPR was defined as the number of pauses greater than two seconds divided by the number of words.

**Result:**

The research study revealed a distinct pattern, with LPR being highest with AD (mean: 0.02), followed by aMCI (mean: 0.01), and nearly zero with normal controls (AD vs aMCI: p = 0.033; aMCI vs Normal: p<0.001). There was also positive correlation between LPR and CDR‐SB (p<0.001) as well as other cognitive tests such as MMSE (p = 0.001) and CASI (p = 0.003), and negative correlation between LPR and hippocampus volume (p<0.001). In multiple regression analysis, our study further showed that LPR alone, could effectively predict CDR‐SB and hippocampus volume (CDR‐SB: β = 57.01, t = 2.93; hippocampus volume: β = ‐21.90, t = ‐3.63). However, no association between LPR and amyloid burden has been found.

**Conclusion:**

Long pause ratio appears rarely in normal controls of our participants. However, LPR increases as the severity of dementia worsens. Moreover, it also correlates with hippocampal size in our cohort. LPR should be considered as a critical linguistic feature in speech analysis.